# Association of DNA-Methylation Profiles With Immune Responses Elicited in Breast Cancer Patients Immunized With a Carbohydrate-Mimicking Peptide: A Pilot Study

**DOI:** 10.3389/fonc.2020.00879

**Published:** 2020-06-05

**Authors:** Cinthia Violeta Hernandez Puente, Ping-Ching Hsu, Lora J. Rogers, Fariba Jousheghany, Eric Siegel, Susan A. Kadlubar, J. Thaddeus Beck, Issam Makhoul, Laura F. Hutchins, Thomas Kieber-Emmons, Behjatolah Monzavi-Karbassi

**Affiliations:** ^1^Department of Pathology, University of Arkansas for Medical Sciences, Little Rock, AR, United States; ^2^UnivLyon, Université Claude Bernard Lyon 1, Lyon, France; ^3^Department of Environmental and Occupational Health, University of Arkansas for Medical Sciences, Little Rock, AR, United States; ^4^Winthrop P. Rockefeller Cancer Institute, University of Arkansas for Medical Sciences, Little Rock, AR, United States; ^5^Division of Medical Genetics, University of Arkansas for Medical Sciences, Little Rock, AR, United States; ^6^Department of Biostatistics, University of Arkansas for Medical Sciences, Little Rock, AR, United States; ^7^Highland Oncolgy Group, Fayetteville, AR, United States; ^8^Division of Hematology Oncology, Department of Internal Medicine, University of Arkansas for Medical Sciences, Little Rock, AR, United States

**Keywords:** DNA methylation, breast cancer, cancer vaccine, immune response, pilot study

## Abstract

Immune response to a given antigen, particularly in cancer patients, is complex and is controlled by various genetic and environmental factors. Identifying biomarkers that can predict robust response to immunization is an urgent need in clinical cancer vaccine development. Given the involvement of DNA methylation in the development of lymphocytes, tumorigenicity and tumor progression, we aimed to analyze pre-vaccination DNA methylation profiles of peripheral blood mononuclear cells (PBMCs) from breast cancer subjects vaccinated with a novel peptide-based vaccine referred to as P10s-PADRE. This pilot study was performed to evaluate whether signatures of differentially methylated (DM) loci can be developed as potential predictive biomarkers for prescreening subjects with cancer who will most likely generate an immune response to the vaccine. Genomic DNA was isolated from PBMCs of eight vaccinated subjects, and their DNA methylation profiles were determined using Infinium^®^ MethylationEPIC BeadChip array from Illumina. A linear regression model was applied to identify loci that were differentially methylated with respect to anti-peptide antibody titers and with IFN-γ production. The data were summarized using unsupervised-learning methods: hierarchical clustering and principal-component analysis. Pathways and networks involved were predicted by Ingenuity Pathway Analysis. We observed that the profile of DM loci separated subjects in regards to the levels of immune responses. Canonical pathways and networks related to metabolic and immunological functions were found to be involved. The data suggest that it is feasible to correlate methylation signatures in pre-treatment PBMCs with immune responses post-treatment in cancer patients going through standard-of-care chemotherapy. Larger and prospective studies that focus on DM loci in PBMCs is warranted to develop pre-screening biomarkers before BC vaccination.

**Clinical Trial Registration:** www.ClinicalTrials.gov, Identifier: NCT02229084.

## Background

We have developed and are clinically testing a cancer vaccine that targets tumor-associated carbohydrate antigens (TACAs), using a carbohydrate-mimicking peptide (CMP) as an immunogen (1, 2). This CMP, called P10s-PADRE, is capable of mimicking the TACAs Lewis Y and ganglioside GD2. These antigens are of interest because of their inherent association with cell survival pathways (3–6). The CMP P10s mimics Lewis Y and GD2 antigens through a shared minimum building block which is the Galβ(1,4)GlcNacβ block in LeY and the lacto-ceramide core (Galβ(1,4)Glcβ(1-1′)Cer) in GD2. We completed a Phase I clinical trial of the P10s vaccine in breast cancer patients and showed its feasibility, safety and immune efficacy. The data indicate induction of anti-peptide and anti-glycan antibodies (1). Antibodies of immunized subjects mediated cytotoxicity on breast cancer cell lines but had no effect on normal breast cell line MCF-10A (1). After more than 7 years of follow up of the phase I clinical trial, four out of six vaccinated subjects are still alive with three of them in stable condition and one subject in remission. In a cancer vaccine trial, generation of an immune response is a prerequisite of clinical efficacy. Therefore, there is an urgent need to identify biomarkers that will allow us to prescreen subjects who are more likely to respond.

Epigenetic changes play crucial roles in tumor development and progression. Aberrant DNA methylation exerts significant impact on the regulation of genes involved in tumorigenesis and tumor progression (7–11). A considerable body of work has focused on DNA methylation at CpG sites (CpGs) in genes associated with BC (12–14). In addition to a direct impact on tumor cells, DNA methylation and histone modifications can affect tumor surveillance by controlling the anti-tumor reactivity of the components of the immune system. Epigenetic mechanisms have been shown to play a critical role during hematopoiesis by contributing to the formation of stable, heritable gene expression patterns (15). T-helper-lineage polarization and cytokine profiles have been associated with differential methylation that, in part, could be due to adaptation to the surrounding conditions (16–20). Several studies have demonstrated that the clonal patterns of expression of killer immunoglobulin-like receptors (KIRs) on NK cells are epigenetically regulated and maintained by DNA methylation (21, 22). Epigenetic mechanisms are also involved in B-cell development (23, 24).

In this study, we sought to determine whether it is possible to define a predictive signature based on differentially methylated loci that can be used to select breast cancer patients who will most likely generate an immune response after vaccination.

## Methods

### Participants and Treatments

This study is part of a single-arm multi-site Phase Ib clinical trial that evaluates the safety, tolerability and feasibility of eliciting adequate IgG response with P10s-PADRE vaccine and MONTANIDE™ ISA 51 VG as an adjuvant. P10s-PADRE peptide synthesis and vaccination was described before (1). This clinical trial was approved by the Institutional Review Board (IRB) of the University of Arkansas for Medical Sciences (UAMS) and was registered with the NIH clinical-trials registry at http://clinicaltrials.gov (NCT02229084). The study was first released on clinicaltrials.gov on 08/26/2014. The IRB was approved on 12/02/2014 and the trial was opened to enrollment on 01/14/2015. The first subject was enrolled on 02/26/2015. Women 18 years of age or older, of all races, with clinical stage I, II or III ER-positive breast cancer were eligible, and subjects were enrolled after providing written informed consent. P10s-PADRE vaccine was given in combination with neoadjuvant chemotherapy in eligible female BC subjects. BC patients were excluded from the trial if they had ER-negative, HER2-positive, inflammatory, metastatic disease, or were pregnant, breast-feeding, with autoimmune diseases, immunosuppressed, or receiving systemic corticosteroids (except those received as antiemetics or for hypersensitivity reactions related to chemotherapy). The patients were recruited from the Breast Cancer Clinic at the Winthrop P. Rockefeller Cancer Institute (WPRCI) at the UAMS campus, and from the Oncology Clinic of the Highlands Oncology Group (HOG) in Northwest Arkansas. For this study, we considered four different vaccination schedules, named A, B, C, and D, that evaluated the feasibility of eliciting an adequate immune response when the vaccine was administered with neoadjuvant chemotherapy. Each schedule had five subjects enrolled. The patients were immunized by administration of three injections of P10s-PADRE vaccine over a 3-weeks period at a dose of 500 μg in a final volume of 2 mL (1 mL peptide and 1 mL of adjuvant) per subcutaneous injection. Patients receiving neoadjuvant chemotherapy were administered doxorubicin (60 mg/m^2^) and cyclophosphamide (600 mg/m^2^) every 3 weeks for four cycles followed by docetaxel (75 mg/m^2^) every 3 weeks for four cycles. The timing of immunizations relative to chemotherapy and blood draws used for antibody and IFN-γ detection for each schedule is shown in Supplementary Table S1.

The subjects selected for this study were selected according to the antibody titers elicited after the 3rd immunization, in which two patients were selected per group: one with the lowest and another with the highest fold increase in IgG titer level (Table 1). Therefore, we sampled each schedule and the samples include subjects with a range of antibody response. De-identified samples were used to generate data and for data analysis. No patient identifiable information was used.

**Table 1:** Patients selected for the study according to their IgG titer response elicited after the third vaccination.

**Group**	**Subject number**	**Fold increase in IgG titer**	Fold increase in IFN-γ levels^*^	**Tumor stage (Tumor grade) at the baseline**	**Tumor size (cm) at the baseline**
A	A3	64	3.97	IIB (3)	2.6
A	A4	1	6.61	IIA (2)	2.5
B	B1	1	16.8	IIA (3)	3.7
B	B3	16	3.6	IIA (2)	3.3
C	C1	32	1	IIIB (1)	7.0
C	C2	4	1.79	IIB (2)	3.7
D	D1	4	1	IIB (2)	4.9
D	D3	16	1	IA (2)	4.6

* *IFN-γ fold change values were log_2_-transformed and analyzed using one-sample t-test. The fold increases among the 8 samples had a mean ± SE of 4.47 ± 1.89 (two-tailed P = 0.030)*.

### Serum Collection and ELISA

Pre- and post-immunization blood was drawn for serum collection from each subject (Supplementary Table S1) and stored at −80°C. For each subject, the ELISA assay was performed on all collected sera at once. Serum samples for each subject were thawed and anti-P10s-IgG levels were measured via ELISA as described earlier (1, 2). The endpoint antibody titers were determined by measuring reactivity of 2-fold serial dilutions of both pre- and post-immune serum, and the fold change in anti-P10s-MAP IgG titer was determined in post-immune serum compared to baseline pre-immune serum. Antibody titer reached its peak starting 4 weeks after the third immunization and remained stable for several weeks as reported before (1). Post-immune sera from weeks 10 or 11 (depending on the treatment schedule) were used to determine fold increase in antibody titer.

### IFN-γ Measurement

Serum IFN-γ levels of the selected subjects were measured using the Meso Scale Discovery^®^ (MSD^®^) multi-spot V-PLEX^®^ assay system (Meso Scale diagnostics LLC, Rockville, MD) according to manufacturer's instructions as a part of the efforts to determine the levels of multiple cytokines in the serum samples collected. The proinflamatory panel 1 (human) kit, which includes IFN-γ, IL-1β, Il-2, IL-4, IL-6, IL-8, IL-10, IL-12p70, IL-13, and TNF-α, was used for cytokine measurements. Briefly, aliquots of serum samples were thawed, diluted 1:2 with Diluent 2 (V-PLEX human Pro-inflammatory Panel 1 kit Cat. # K15049D, MSD), and added to the V-PLEX plates along with standards. The plates were shaken at 700 rpm and incubated overnight at 4°C. After washing the plates 3 times with phosphate-buffered saline plus 0.05% Tween-20 (PBS-T), 25 μL of detection antibody was added to each well. The plate was then placed on a plate shaker and mixed at 700 rpm for 2 h at room temperature. Following incubation, the plate was washed 3 times with PBS-T and 150 μL of 2X Read Buffer was added to each well. The plate was analyzed using the MSD Electrochemiluminescence (ECL) charge-coupled device (CCD) and QuickPlex SQ 120 imager and the results were reported using the Discovery Workbench 4.0 software (MSD). The pre-immune (collected at week 1) and post-immune sera (collected at weeks 7 or 8, depending on the treatment schedule, see Supplementary Table S1) were used for IFN-γ measurement. Subject A3 had its week 7 blood missing and therefore the week 10 serum of this subject was used to determine the post-immune cytokine levels. The fold increase in IFN-γ levels for each subject was determined in post-immune serum compared to baseline pre-immune serum.

### Isolation of Peripheral Blood Mononuclear Cells (PBMCs)

Forty milliliters of freshly collected blood was used to isolate PBMCs. The centrifuge and all reagents were brought to room temperature for at least an hour prior to PBMC isolation. Histopaque-1077 (Sigma) in a volume equal to the volume of collected blood was transferred into 50-ml conical centrifuge tube. Then, blood was carefully layered onto the Histopaque-1077 at a ratio of 1:1. The mixture was then centrifuged at 400 *g* at room temperature for 30 min. Following centrifugation, all the plasma was aspirated and transferred to other tubes and 0.5 cm of the opaque interface containing mononuclear cells was collected into a clean 50-ml conical centrifuge tube. The cells were washed by adding PBS to a total volume of 45–50 ml. The suspension was then centrifuged at 250 *g* for 10 min at room temperature. After discarding the supernatant, this washing step was repeated two more times to ensure the removal of debris. Finally, the cell pellet was frozen and stored in liquid nitrogen until use.

### DNA Isolation and DNA Methylation Analysis

Pre-immune PBMC samples from select subjects were thawed and DNA was isolated using the Gentra Puregene Cell Kit (Qiagen Inc., Valencia, CA) according to the manufacturer's protocol. Briefly, approximately 1.5 million cells were lysed and processed to remove cellular byproducts. DNA was isolated by ethanol precipitation to a volume of 100 μL. The samples were quantified using Quant-iT PicoGreen dsDNA Assay Kit (Thermo Fisher, Waltham, MA), and diluted in TE buffer (Sigma Aldridge, St. Louis, MO) to a concentration of 20 ng/μL in 40 μL (800 ng). 500 ng of genomic DNA from each patient sample was bisulfite-treated and purified using the EZ DNA Methylation-Gold kit (Zymo Research, Irvine, CA) according to the manufacturer's instructions. Genome-wide DNA methylation was assessed in bisulfite-converted genomic DNA using the Infinium MethylationEPIC BeadChip array (Illumina, San Diego, CA), following the Infinium HD Methylation Assay Protocol User's Guide provided by Illumina. Processed BeadChips were scanned on an Illumina iScan^®^, and methylation values were determined for all probes using the GenomeStudio Methylation module (Illumina).

### Analyses of DNA Methylation Data

Illumina intensity data (.idat) files from the chip were extracted using the Methylation module (v.1.8.0) of the GenomeStudio (v.2011.1) software from Illumina. CpG probes with a detection *p* > 0.01 and rsSNPs were removed using this software. DNA methylation levels were reported as β values, which are measurements of the degree of methylation at a specific CpG locus that range from zero (0%) to one (100%), where zero indicates a non-methylated probe and one indicates a fully methylated probe. The .idat files were transferred into Partek Genomics Suite (St. Louis, MO) and probes located in the X and Y chromosomes and those with polymorphic targets and cross-hybridization potential [non-specific probes described on (25)] were filtered out. At the end of all filtrations, we had a total of 527,362 CpG probes that were functionally normalized. Finally, the β-values of the filtered CpGs were transformed to M-values by using this equation: M-value = log_2_ (β/(1–β)), and the M-values were used for the statistical analysis.

The identification of differentially methylated CpGs (DM CpGs) among responders and non-responders to P10s-PADRE IgG response was done via Method of Moments using the following linear regression model applied to each CpG site: Y_*i*_ = μ + log_2_ (fold change in Ab titer) + ε_*i*_, Y_*i*_ represents the M-value of the *i*th subject, μ the intercept, fold change in Ab titer the independent variable and ε_*i*_ the error term. Moreover, through an alternative analysis, we correlated the subjects' methylomes to their IFN-γ levels using the same statistical model with fold change in IFN-γ concentration of serum substituted for fold change in Ab titer, as follows:

$${\text{Y}}_{i}\;=\text{ μ }+\;{\text{log}}_{\text{2}}\;(\text{fold change in IFN-γ concentration})\text{ + }{\text{ε}}_{i}.$$

A CpG site was identified as DM with respect to either Ab titer or IFN-γ levels if it was statistically significant in the linear regression model with either log_2_ (Ab titer) or log_2_ (IFN-γ), respectively, as independent variables. Statistical significance was defined using an alpha ≤ 0.001 significance level to adjust for the multiple CpG sites being tested without overinflating Type II (false-negative) error in this small-scale pilot study.

### Pathway and Network Analyses

In order to interpret the BeadChip array data, we used Ingenuity^®^ Pathway Analysis (IPA^®^) tool, version 01–12. IPA is based on the Ingenuity Knowledge Base that contains manually curated content related to studies and observations from biomedical literature and also integrated from other data bases (26). The lists of DM CpGs and their respective *p*-values were uploaded as excel files into IPA, and by using the Core Analysis, the canonical pathways and networks associated to them were developed. The selected pre-analysis parameters were: file format selected as flexible, the reference dataset was Illumina Human Methylation 450 v1-v2, the relationships to consider were direct and indirect based on published interactions, all the nodes (molecules) were selected, the number of molecules per network was 35, the confidence chosen was experimentally observed (not predicted), information associated to tissues and cell lines and the species was human.

The networks are shown as genes or their products (nodes) and their biological relationships (edges). Networks contain a score based on the number of eligible molecules they contain. The score is calculated through Fisher's Exact Test. The higher the score, the probability to find the molecules present on the network by random chance is lower and considering this, the network with the highest score was the one selected for each analysis. The same test is used for the pathways' discovery. The *p*-value in the canonical pathways depends on the size of the pathway and is less significant when the proportion of the reference set molecules present in the pathway is larger. Another measurement is the ratio and it is estimated by considering the number of genes from the uploaded data set that are on the canonical pathway and is divided by the total number of genes present in the pathway, hence the ratio shows the percentage of genes in a given pathway that were also present in the uploaded gene list.

## Results

### Association of Differentially Methylated Loci With Antibody Levels in Immunized Subjects

**Figure 1:**
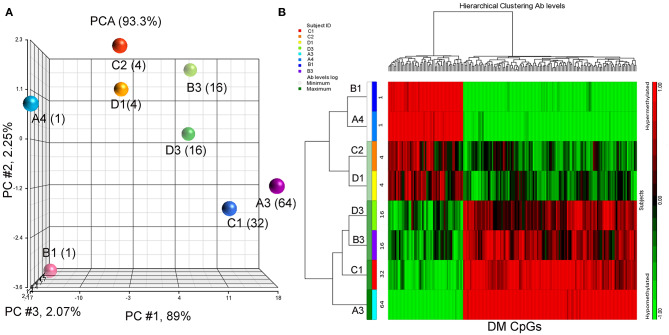
PCA plot and heatmap based on the 182 differentially methylated CpG loci (*p* ≤ 0.001) from the regression model considering IgG titers. **(A)** PCA scatter plot demonstrates separation according to the anti-peptide IgG levels. **(B)** Heatmap representing the similarities of DNA methylomes clustered according to their IgG levels (in fold change). The columns represent the CpG probes and rows the samples taken from indicated subjects. CpG probes with no changes in methylation have a value of zero and colored in black. Hypermethylated probes are displayed in red and hypomethylated probes in green. Subjects' study-numbers and fold increases in anti-peptide antibody titers are shown.

Eight BC patients (Table 1) were included in the present study. The anti-P10s antibody titer was measured in pre- and post-immune sera (Supplementary Table S1) and the fold change in serum antibodies in post-immune serum compared to pre-immune serum was determined (Table 1). Global DNA methylation was performed using the Infinium Human MethylationEPIC BeadChips in pre-immunized PBMCs collected from each subject. The data were processed after filtering undesirable probes (see methods), and the analysis proceeded with 527,362 probes. Using a linear regression model, we assessed the association between DNA methylation and IgG levels elicited after the third vaccination with P10s-PADRE. 182 DM CpGs were identified among the subjects (*P* ≤ 0.001), and 135 of them mapped into annotated genes as shown in Supplementary Table S2. Partial correlation coefficients, sum of squares and F values are provided for the 182 CpG probes (Supplementary Table S2). The correlation line for Top 3 genes suggest a significant relationship between methylation levels and fold change in antibody response (Supplementary Figure S1). Principal-component analysis (PCA) using the 182 DM CpGs distinguished the methylomes of vaccinated subjects according to their anti-peptide IgG levels (Figure 1A). From Figure 1A, the first principal component (PC #1 axis) explains 89% of the variation and clearly separates subjects according to their antibody fold change. Hierarchical clustering produced methylation signatures of those DM CpGs according to anti-peptide IgG levels (Figure 1B). Thus we could note two groups in one cluster formed by methylomes from non-responders (A4, B1) and low antibody titers responders (C2, D1: 4-fold) indicating their methylation signatures are more similar in comparison to those from the patients with higher IgG levels: 16-, 32-, and 64-folds which were clustered together, although methylomes of subjects C1 and A3 (32- and 64-folds, respectively) were separated clades.

**Figure 2:**
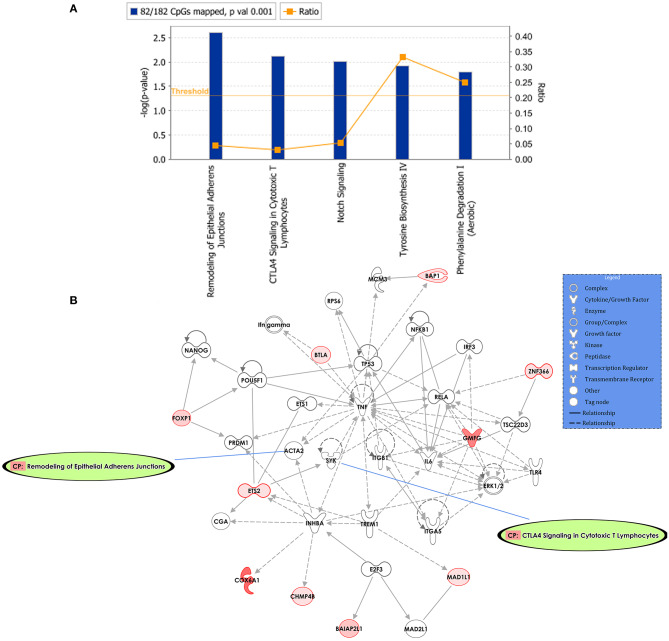
Potential biological roles of the differentially methylated genes (*p* ≤ 0.001) from the regression model considering IgG titers. **(A)** Affected canonical pathways based on the differentially methylated genes in the study. The line defining threshold with a score of 1.3 represents the -log (*p*-value 0.05) on the y-axis of the bar chart. The height of the bar is related to the significance on the overlap of the analyzed genes with the pathway, and the ratio indicates the number of uploaded genes over the total number of genes involved in each of the pathway. **(B)** Top network and overlay of two related canonical pathways. The network is related to cell death and survival, cell cycle, and cellular development. Nodes (genes) and edges (gene relationships) are described in the legend. The intensity of the node color (red) denotes the degree of upregulation (redder is more significant according to the *p-*value). Uncolored nodes were integrated into the network based on evidence stored in the Ingenuity Knowledge Base.

Predicted interactions of the differently regulated genes were generated by IPA using the dataset of the 182 DM CpG profiles and their respective *P-*values. Eighty-two out of 182 DM CpGs mapped into the IPA database and were significantly enriched in five affected canonical pathways, including: remodeling of epithelial adherens junctions, CTLA4 signaling in cytotoxic T lymphocytes, NOTCH signaling, tyrosine biosynthesis IV and phenylalanine degradation I (Figure 2A, Table 2). Ten focused molecules from this study were mapped in the top network that was associated with cell death and survival, cell cycle, and cellular development (Figure 2B). Detailed annotations of the network can be found in Supplementary Table S3.

**Table 2:** Top canonical Pathways and network.

**Name**	***p*-value**	**Overlap**	**DM CpGs in the pathways**
Remodeling of epithelial adherens junctions	2.46 × 10^−3^	4.5% 3/66	ARPC3, CBLL1, TUBB4A
CTLA-4 signaling in cytotoxic lymphocytes	7.45 × 10^−3^	3.1% 3/98	AP1S3, AP2M1, LAT
Notch signaling	9.91 × 10^−3^	5.4% 2/37	DLL4, NOTCH4
Tyrosine biosynthesis IV	1.21 × 10^−2^	33.3% 1/3	PCDB1
Phenylalanine degradation I (Aerobic)	1.62 × 10^−2^	25% 1/4	PCDB1
**Associated network functions**			**Score**
Cell death and survival, cell cycle, cellular development			17

### Association of Differentially Methylated Loci With IFN-γ Levels Elicited After Immunization

We measured levels of cytokines IFN-γ, IL-1β, Il-2, IL-4, IL-6, IL-8, IL-10, IL-12p70, IL-13, TNF-α in pre- and post-immune serum samples using a validated multiplex assay. We observed reliable mean fold increases [Mean (±SE)] in IL-6 (1.64 (±0.22), *P* = 0.03), IL-10 (2.23 (±0.61), *P* = 0.048), and IFN-γ (4.47 (±1.89), *P* = 0.03). *P*-values arise from two-tailed one-sample *t*-tests of log_2_ transformed data.

**Figure 3:**
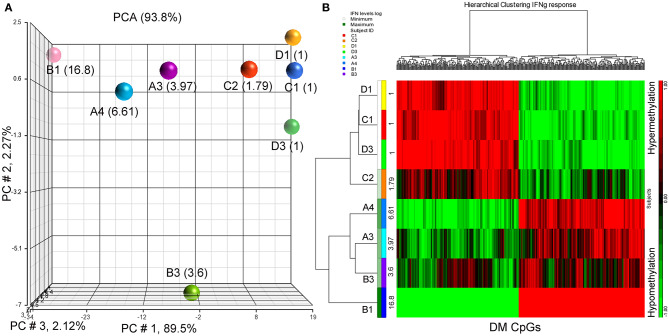
PCA plot and heatmap based on the 398 differentially methylated CpG loci (*p* ≤ 0.001) from the regression model considering IFN-γ levels. **(A)** PCA scatter plot demonstrates that DNA methylation signatures of the subjects are different according to their IFN-γ levels. **(B)** Heatmap representing the similarities of DNA methylomes clustered according to their cytokine levels. The columns represent the CpG probes and rows the samples taken from indicated subjects. CpG probes with no changes in methylation have a value of zero and colored in black. Hypermethylated probes are displayed in red and hypomethylated probes in green. Subjects' study-numbers and fold increases in IFN-γ levels are shown.

Since IFN-γ plays an important role in tumor immune surveillance (27), we also used fold increase in IFN-γ levels in post-immune serum samples to study the association with the subjects' methylomes. Using the same analysis approach employed previously with the addition of log_2_ (fold increase in IFN-γ level) as the independent variable, 398 DM CpGs were identified (*P* ≤ 0.001), of which 324 were mapped into annotated genes (Supplementary Table S4). Partial correlation coefficients, sum of squares and F values are provided for the 324 CpG probes (Supplementary Table S4). The correlation line for Top 3 genes suggest a significant relationship between methylation levels and fold change in IFN-g levels response (Supplementary Figure S2). Through a PCA scatter plot (Figure 3A), the separation of the subjects with the lower levels of IFN-γ change (1-fold: C1, D1, D3 and 1.79-fold: C2) compared to the other subjects who had higher levels (A3, B3, A4, and B1: 3.97-, 3.6-, 6.61-, and 16.8-fold, respectively) was observed on PC # 1, which explains 89.5% of the variation (Figure 3A). Through hierarchical clustering, subjects with low IFN-γ levels (1- and 1.79-folds) were grouped together while those with higher IFN-γ levels were clustered separately (Figure 3B).

**Figure 4:**
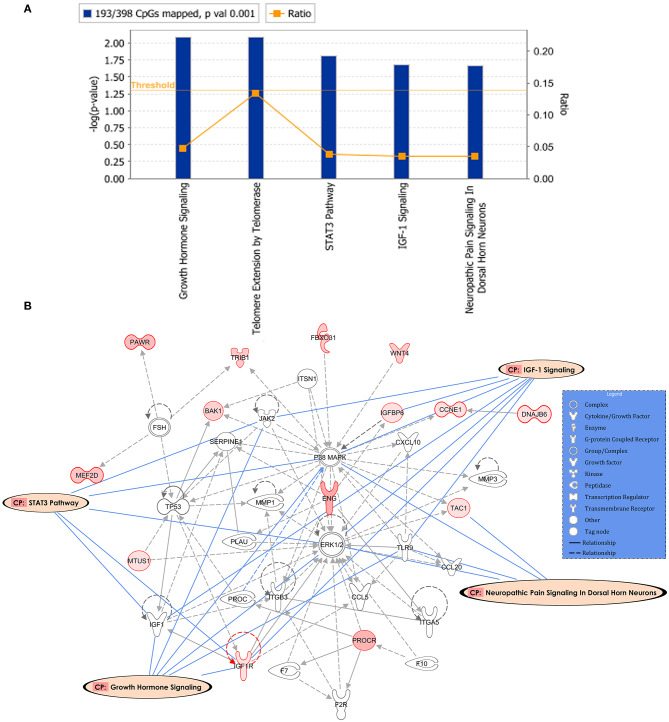
Potential biological roles of the differentially methylated genes (*p* ≤ 0.001) from the regression model considering IFN-γ levels. **(A)** Coloring and display are as in Figure 2A. **(B)** Top network and overlay of four related canonical pathways. The network is related to cellular movement, cell death and survival, cell-to-cell signaling and interaction. Coloring and display are as in Figure 2B.

Following the same strategy of the previous analysis, 398 DM CpGs were uploaded into IPA and 193 out of 398 DM CpGs were mapped into the IPA database. The top five canonical pathways affected by IFN-γ levels were found, including growth hormone signaling, telomere extension by telomerase, STAT3 pathway, IGF-1 signaling and neuropathic pain signaling in dorsal horn neurons (Figure 4A, Table 3). Fourteen focused molecules were mapped to the top network that was associated with cellular movement, cell death and survival, cell-to-cell signaling and interaction, with four of the canonical pathways mentioned above as shown in Figure 4B. Supplementary Table S5 includes the gene network annotations.

**Table 3:** Top canonical Pathways and network identified.

**Name**	***p*-value**	**Overlap**	**DM CpGs in the pathway**
Growth hormone signaling	8.16 × 10^−3^	4.7% 4/85	GHR, IGF1R, PIK3C2A, SOCS6
Telomere extension by telomerase	8.34 × 10^−3^	13.3% 2/15	POT1, TNKS
STAT3 Pathway	1.57 × 10^−2^	3.9% 4/103	GHR, IGF1R, SOCS6, TNFRSF11A
IGF-1 Signaling	2.07 × 10^−2^	3.6% 4/112	IGF1R, IGFBP6, PIK3C2A, SOCS6
Neuropathic pain signaling in dorsal horn neurons	2.19 × 10^−2^	3.5% 4/114	GRM8, KCNQ2, PIK3C2A, TAC1
**Associated network functions**			**Score**
Cellular movement, cell death and survival, cell-to-cell signaling and interaction			20

## Discussion

Cancer vaccines, whose goal is to elicit an active induction of anti-tumor response, are emerging strategies for cancer treatment. We are in phase Ib of a clinical trial testing a vaccine targeting TACAs. A major obstacle in targeted active immunotherapies is that some subjects do not experience an immune response to the vaccine. Prescreening to identify likely responders not only improves the robustness of clinical trials, it also improves participation rates among candidates. Furthermore, prescreening to select a responder population becomes necessary as a vaccine moves toward phase II/III clinical trials and commercialization.

DNA methylomes have been correlated with immune response to vaccination against influenza (28–30) and with immune response after BCG vaccination (31, 32), using PBMCs. DM loci were also related to BC progression, and have shown utility in stratification of disease stages, as well as in distinguishing normal tissue from cancer tissue (9, 12, 33–35). In this study, we examined the methylome in baseline PBMCs of BC subjects prior to vaccination and chemotherapy to determine whether DM loci correlate with vaccination outcome and can be used as predictive markers of immune responses.

PCA and hierarchical clustering analyses showed that antibody response correlates with methylation differences in our patient population. The analysis distinguished responders with similar methylation patterns. In a parallel analysis using IFN-γ levels as the immune response endpoint, PCA analysis separated subjects by the amount of fold increase of this serum cytokine after vaccination, indicating that the subjects share similar methylation signatures that impact the magnitude of their immune response.

To further understand how these DM loci could affect immune response to the vaccine, analyses of pathways and molecular networks impacted by DM loci (36, 37) were performed by IPA based on Ab response levels among subjects. Five top altered canonical pathways were identified, with remodeling of epithelial adherens junctions being the most affected canonical pathway. These junctions are key factors for maintenance of epithelial homeostasis, regulation of cell migration, and tumor progression, and are associated with tumor invasion and progression (38, 39). Moreover, cell-cell junctions play a role in cross-talk with immune cells. For instance, E-cadherin can participate in adaptive immunity through its involvement in the immunogenicity of dendritic cells (DCs) (40, 41). In addition, E-cadherin ligands are expressed on T-cells and NK cells that mediate their direct interaction with E-cadherin-expressing cells (42, 43). Additionally, Epithelial-to-mesenchymal transition (EMT) in tumor cells is marked by downregulation of E-cadherin and is linked to immune escape mechanisms (44).

CTLA-4 signaling in cytotoxic lymphocytes also showed significant DM pathway among the subjects, suggesting that the status of controllers of immune homeostasis may play a role in induction of the IgG response. An interesting DM locus from that pathway was AP2M1, which interacts with CTLA-4 in regulating its internalization and cell surface expression (45), affecting the activation and proliferation of T cells (46, 47). Moreover, LAT, another gene related to the T-cell activation, development and survival (48) was significantly differentially methylated in our study. Additionally, two Notch signaling pathway related genes were differentially methylated in our analysis: DLL4 and NOTCH4. Notch pathway regulates T helper 1 (Th1) activation, maintains memory T cells, and upregulates the expression of granzyme B and perforin that can augment the cytotoxic function of immune effector cells. Moreover, this pathway is essential for type 1 innate lymphoid cells and conventional NK cells polarization and functions (49). Notch has a key role in the regulation of immune cell differentiation and their functions, presenting it as an important therapeutic target as in the case of DLL4+ DCs that have the potential to increase the antitumor immunity (50–52).

When IFN-γ levels as the immune response endpoint was considered, the top network identified by IPA was related to cellular movement, cell death and survival, cell-to-cell signaling and interaction and it overlapped with the STAT3, growth hormone (GH) and IGF-1 signaling pathways. GH signaling plays a key role in cancer development and its activation can lead to STAT1, STAT3 and STAT5 signaling (53). The IGF-1 signaling pathway, another pathway identified, is directly related to GH and STAT3 signaling and is also associated with drug resistance in ER+ BC patients (54, 55).

Cancer-related methylation changes are detectable in the DNA of blood cells (56), suggesting cancer-associated aberrant methylation can be confounded with immune parameters. The confounding could be due to factors like tumor stage, the status of metastatic disease (57), chemotherapy received (58, 59), age (60), obesity (61), or other comorbidities. It is also possible that a pathway associated with cancer progression can also affect immune response. Thus, more comprehensive studies are needed to make unbiased conclusions. We did not study gene expression and its correlation with methylation profile in this pilot study. Future studies are needed to further establish biological significance of differentially methylated genes.

The data presented herein demonstrate a complex interrelationship between pathways involved in immune system, as well as signaling directly affecting tumor progression. However, methylation patterns in baseline PBMCs are associated with post-treatment immune responses that indicate the feasibility of developing predictive signatures based on DM loci to prescreen breast cancer patients who will more likely generate an immune response after therapy. The data warrant further investigation in larger cohorts.

## Data Availability Statement

The original contributions presented in the study are publicly available. This data can be found here: the European Genome-Phenome Archive (EGA, https://ega-archive.org) which is hosted at the EBI and the CRG, under study number EGAS00001004211 with accession number EGAD00010001854.

## Ethics Statement

The studies involving human participants were reviewed and approved by Institutional Review Board of the University of Arkansas for Medical Sciences (reference number 202556), and registered with the NIH clinical-trials registry at http://clinicaltrials.gov (NCT02229084). The patients/participants provided their written informed consent to participate in this study.

## Author Contributions

CH, P-CH, SK, and BM-K performed full methylation analysis and summarized data. FJ, LR, and BM-K conducted the laboratory experiments. ES conducted the statistical analysis. LH, IM, ES, SK, BM-K, and TK-E designed the study. JB, LH, and IM conducted the clinical trial and along with the other authors contributed to the writing of the manuscript.

## Conflict of Interest

TK-E and BM-K are named as inventors on an institutional patent application filled by UAMS that is related to the content of this manuscript. Therefore, TK-E, BM-K, and UAMS have a potential financial interest in the vaccine used in this clinical trial. No financial or other support of any kind has resulted from this patent application. These financial interests have been reviewed and the clinical trial was performed by approved supervision in accordance with the UAMS conflict of interest policies. The remaining authors declare that the research was conducted in the absence of any commercial or financial relationships that could be construed as a potential conflict of interest.

## Abbreviations

BC: breast cancer
CMP: carbohydrate-mimicking peptide
DM: differentially methylated
GD2: disialoganglioside 2
IFN-γ: interferon gamma
IgG: immunoglobulin G
IRB: Institutional Review Board
LeY: Lewis Y antigen
mAb: monoclonal antibody
NK: Natural killer
PADRE: pan-DR T-helper epitope
PBMCs: peripheral blood mononuclear cells
PCA: principal-component analysis
SC: subcutaneous
SE: standard error
TACA: tumor-associated carbohydrate antigens
Th1: T-helper 1
UAMS: University of Arkansas for Medical Sciences.
