# Supplementary material for: Association of DNA-Methylation Profiles With Immune Responses Elicited in Breast Cancer Patients Immunized With a Carbohydrate-Mimicking Peptide: A Pilot Study
Source: Front Oncol. 2020 Jun 5;10:879. doi: 10.3389/fonc.2020.00879 (PMC7290046; doi:10.3389/fonc.2020.00879)
Supplement: Supplementary file 6 [file Data_Sheet_1.DOCX]

## Supplementary Figures


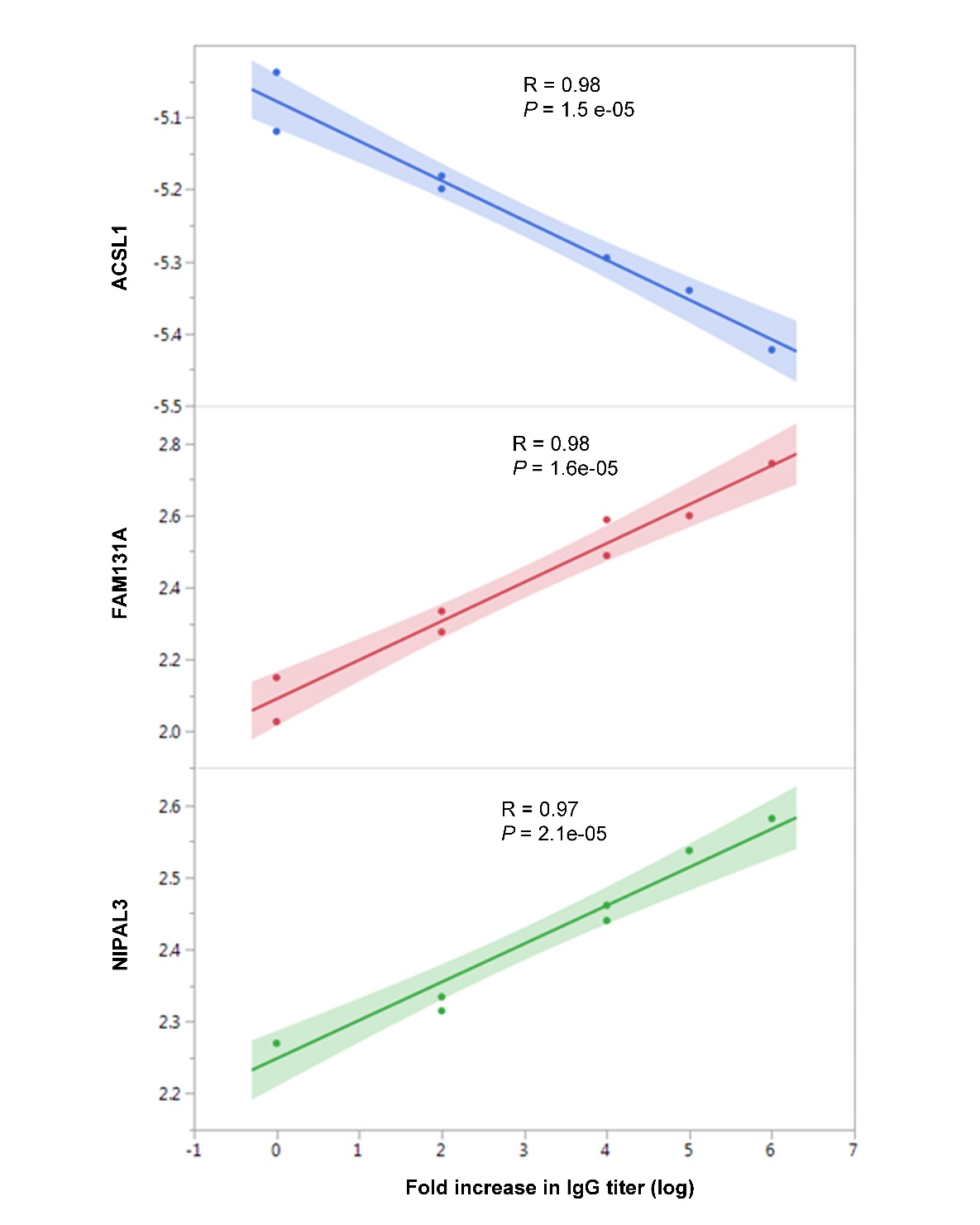


**Supplementary Figure 1.**.  Correlation line and 95% CI between fold increase in antibody levels generated in post-immune serum and methylation levels in the pretreated PBMCs for the top 3 genes from Table S2 is depicted . Correlation coefficient (R) and p value for each correlation line are shown.


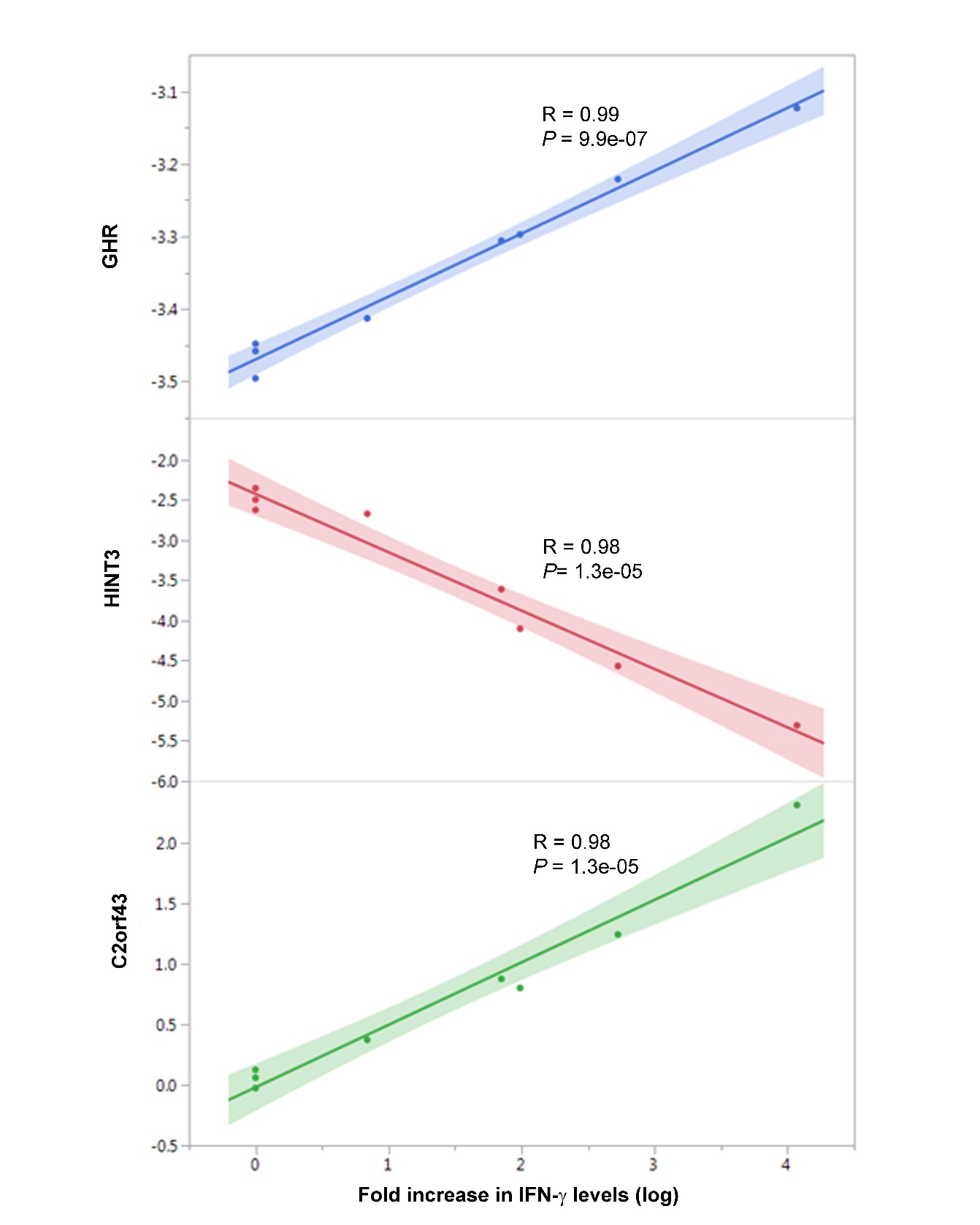


**Supplementary Figure 2**.  Correlation line and 95% CI between fold increase in IFN-γ levels generated in post-immune serum and methylation levels in pretreated PBMCs for the top 3 genes from Table S4 is depicted. Correlation coefficient (R) and p value for each correlation line are shown.
